# Exact Response Theory for Delay Equations

**DOI:** 10.3390/e28030350

**Published:** 2026-03-20

**Authors:** Federico Gollinucci, Enrico Ortu, Lamberto Rondoni

**Affiliations:** 1 Department of Mathematical Sciences “Giuseppe Luigi Lagrange”, Politecnico di Torino, 10129 Torino, Italy; federico.gollinucci@polito.it (F.G.); enrico.ortu@polito.it (E.O.); 2 Istituto Nazionale di Fisica Nucleare, Sezione di Torino, Via P. Giuria 1, 10125 Torino, Italy; 3 CONCEPT Lab, Fondazione Istituto Italiano di Tecnologia, Via E. Melen 83, 16152 Genova, Italy

**Keywords:** Transient Time Correlation Function, uncertainty quantification, dissipation function

## Abstract

The exact response theory, also known as Transient Time Correlation Function formalism, is a powerful method concerning how observables respond to a given perturbation of the dynamics of the systems of interest, and it extends linear response theory to generic (autonomous) dynamical systems. Its main ingredient is the so-called dissipation function. In this paper, we adapt this theory for time-lagged systems, and we illustrate its applicability considering simple examples of delay equations, with different memory terms. Adopting the technique already used for time deterministic as well as stochastic time-dependent perturbations, the dynamics is described in a higher dimensional phase space, in which the delay-dependent dynamics is mapped into an augmented phase space: the new dynamics is proven to be autonomous and suitable for the exact responses to be computed. In addition, we explore the comparison between linear and exact approaches for a specific kernel choice.

## 1. Introduction

One of the most successful tools developed in statistical mechanics is linear response theory [1], which is an easy and intuitive approach to describe macroscopic systems driven away from, but close to, thermodynamic equilibrium in terms of their equilibrium dynamics and probability distributions [2,3]. Its range of applicability is wide and widely used in a lot of fields, such as climate physics [4], and justifies all linear transport laws. However, it applies to small drivings and away from critical situations such as those concerning phase transitions. More recently, an exact response theory has been developed within the field of molecular dynamics, emerging from the literature concerning fluctuation theorems [5,6,7]. This theory is also called Transient Time Correlation Function (TTCF) formalism [8,9,10].

While the linear theory mainly concerns macroscopic objects and their thermodynamic properties [11], the results of this exact response theory are particularly useful in systems strongly driven away from equilibrium and in small systems, which are easily found far from equilibrium. It has also been proven to effectively tackle phase transitions [12] and a rich variety of problems [13,14,15]. Developed for autonomous systems, the theory has been later extended to deterministic time-dependent perturbations [16,17], and then to stochastic time-dependent perturbations [18], as well as to quantum mechanics [19]. A crucial ingredient of the theory is the *dissipation function*, which, in the case of particle systems and under proper conditions, can be identified with the energy dissipation rate, the object of the Fluctuation Theorems [6,20].

In this paper, we propose an adaptation of the methods used in [17,18] for treating systems characterized by a time-delay in the dynamics, which are a fundamental tool of investigation for feedback control [21], and other countless phenomena [22,23,24]. In doing this, we will also touch on the problem of quantification of uncertainties [25], which is of interest in general time-evolving systems well beyond statistical mechanics. This involves the derivation of information on the moments of the distributions under study, which the exact response theory allows, since the powers of an observable are observables themselves [3,26].

The paper is structured as follows. In Section 2, we recap the main ideas behind the exact response theory, or TTCF, starting from the linear response theory, a fundamental framework in which the role of the initial perturbation is highlighted. After that, the augmented phase-space method is summarized, where a time-dependent perturbation is described within an enlarged phase space: its role is to embed both the physical dynamics and the time-dependence of the perturbation within a harmonic oscillator. This choice allows one to describe the system in the higher-dimensional space in terms of an autonomous dynamics, in which the time-independent theory can be applied. In Section 3, we develop the model of a particle within a viscous media whose effect on the velocity is time-delayed. Specifically, we analyze three kinds of kernels: the Erlang-type kernel, a rich class including a long-tail distribution for the delay [27], the exponential as its special first-order case, and the stepwise kernel. We also analyze how averages of simple quantities change. We computed as examples the averages of some interesting physical quantities. Furthermore, in Section 4 we provide a comparison between the linear response approach and the exact response method. Finally, in Section 5 we discuss our work and summarize the main results of the paper and how the augmented phase-space method allows one to treat time-delayed systems: this idea can be applied to a wide class of problems, as discussed below.

## 2. Response Theory

This section recaps the mathematical background for the response theory and summarizes the main results of [28,29,30], explained in higher detail in [3].

Suppose M is the phase space of a physical system whose quantities are described by the phase-space variable Γ∈M, where $\Gamma \;:=\;(q_{1},\ldots ,q_{n},p_{1},\ldots ,p_{n})$ represents the configuration of a system of *n* particles, and suppose the known evolution rule is defined in terms of the following dynamical system:

(1) $$\dot{\Gamma }=G\left(\Gamma \right),$$

whose solution at time *t*, given by $S^{t}\Gamma_{0}$, concerns the initial condition $\Gamma_{0}$ and the evolution operator $S^{t}$ [31]. A useful quantity is the phase-space variation rate Λ:M→R defined as

(2) $$\Lambda :=\nabla_{\Gamma }\cdot G.$$

where the dot represents the scalar product. Endow the phase space with a suitable probability measure absolutely continuous with respect to the Lebesgue measure, $d\mu_{0}\left(\Gamma \right)\;=\;f_{0}\left(\Gamma \right)\Gamma$, where $f_{0}$ is the probability density. Let a physical observable be defined by O:M→R. If $d\mu_{0}$ is not invariant, its density evolves in time and is denoted by $f_{t}$ at time *t*. Then, the phase-space average of O at time *t* is expressed by

(3) $$E_{f_{t}}\left(O\right)=\int_{M}O\left(\Gamma \right)f_{t}\left(\Gamma \right)d\Gamma .$$

### 2.1. Linear Response Theory

Linear response theory is a powerful tool useful for understanding the effect of a small intensity perturbation. Given a physical system described by the dynamical system $G_{0}\left(\Gamma \right)$, suppose a perturbation is switched on at time t=0, in the form of an additive vector field $G_{ext}\left(\Gamma ,t\right)=F\left(t\right)\pi \left(\Gamma \right)$:

(4) $$G\left(\Gamma ,t\right)=G_{0}\left(\Gamma \right)+G_{ext}\left(\Gamma ,t\right),$$

where *G* governs the dynamics after the perturbation of dimensionless intensity F, and π depends only on the phase variable Γ. The evolution of the probability density in time starting from an equilibrium distribution $f_{0}$ is described in terms of the Liouvillian operator L, which satisfies the Liouville equation:

(5) $$\frac{\partial f}{\partial t}=-\nabla_{\Gamma }\cdot \left(fG\right)=-\nabla_{\Gamma }\cdot \left(fG_{0}+fG_{ext}\right)=-i\left(L_{0}+L_{ext}\right)f=:-iLf$$

where $L_{0}f:=-i\nabla_{\Gamma }\cdot \left(fG_{0}\right)$ denotes the Liouvillian of the unperturbed dynamics, and $L_{ext}f:=-i\nabla_{\Gamma }\cdot \left(fG_{ext}\right)$ the one of the perturbation. Truncating to first order in the perturbation, the solution of the Liouville equation takes the form

(6) $$f_{t}\left(\Gamma \right)=e^{-itL_{0}}f_{0}\left(\Gamma \right)-i\int_{0}^{t}dse^{-i\left(t-s\right)L_{0}}L_{ext}\left(s\right)f_{0}\left(\Gamma \right)+H.O.$$

where the higher orders in F are taken into account by H.O. and thus are negligible. The corresponding linear approximation to the evolution of observables is then expressed by [2,3]

(7) $$E_{f_{t}}\left[O\right]=E_{f_{0}}\left[O\right]+\int_{0}^{t}dsR\left(t-s\right)F\left(s\right),$$

where R(t) is the response function expressed by

(8) $$R\left(t\right)=\beta E_{f_{0}}\left[\left(O\circ S_{0}^{t}\right)J\right],$$

in which *J* is the dissipative flux. This celebrated result, is the basis of the linear transport laws. Remarkably, it shows that memory is practically unavoidable in the response of dynamical systems. This fact can be neglected when describing the behavior of thermodynamic systems, because the memory decays so rapidly compared to observation times that it is not relevant on the macroscopic scale. On the other hand, many non-thermodynamic behaviors are known, even at the macroscopic level, in which memory effects are unavoidable [3].

### 2.2. The Time-Independent Exact Response

The exact response theory was first developed by [7]. In this section, we summarize the time-independent exact response theory in the framework of general dynamical systems, which have been thoroughly explained in [3,9]. Suppose that the dynamics is described by Γ∈M in terms of the following evolution rule:

(9) $$\dot{\Gamma }=G\left(\Gamma ,t\right)=G_{0}\left(\Gamma \right)+G_{ext}\left(\Gamma ,t\right),$$

where $G_{ext}\left(\Gamma ,t\right)$ denotes the perturbation employed on the unperturbed dynamics, with flux $S^{t}\Gamma$. Let $f_{0}$ be a probability density function such that $d\mu \left(\Gamma \right)=f_{0}\left(\Gamma \right)d\Gamma$. Performing the divergence operations in Equation (5), and grouping terms in a different way, the Eulerian form of the Liouville equation (Equation (5)) can also be written as

(10) $$\frac{\partial f}{\partial t}\left(\Gamma \right)=\Omega^{f}\left(\Gamma \right)f\left(\Gamma \right),$$

with $\Omega^{f_{t}}\left(\Gamma \right)$ being known as the dissipation function, defined by

(11) $$\Omega^{f_{t}}\left(\Gamma \right):=-\Lambda \left(\Gamma \right)-G\left(\Gamma \right)\cdot \nabla_{\Gamma }\ln f_{t}\left(\Gamma \right),$$

where Λ is defined in (2). The solution of (10) takes the form

(12) $$f_{s+t}\left(S^{t}\Gamma \right)=\exp\left\{\Omega_{-t,0}^{f_{s}}\left(\Gamma \right)\right\}f_{s}\left(\Gamma \right),$$

where $\Omega_{s,t}^{f_{0}}\left(\Gamma \right)=\int_{s}^{t}\Omega^{f_{0}}\left(S^{\zeta }\Gamma \right)d\zeta$ is the integral of the dissipation function along the trajectory of the flux of Γ between *s* and *t*. As in the linear regime, we are interested in the evolution of the observables rather than the probabilities. In this case, one obtains the following:

(13) $$E_{f_{t}}\left(O\right)=E_{f_{0}}\left(O\right)+\int_{0}^{t}E_{f_{0}}\left[\Omega^{f_{0}}\left(O\circ S^{s}\right)\right]ds,$$

where ∘ denotes the composition, i.e., the evaluation of the observable under the action of the dynamics $O(S^{s}\cdot )$.

Equation (13) highlights that the evolution of the ensemble average of an observable can be computed in terms of the unperturbed equilibrium distribution $f_{0}$: the key difference between the linear case lies in the fact that the exact evolution is given by the dissipation function $\Omega^{f_{0}}$ and that the flow of the dynamical system is the perturbed one $S^{s}$. Originally derived for time-independent perturbations, this theory has then been extended to time-dependent perturbations [16,17], to stochastic time dependencies [18], and to quantum mechanics [19]. In the next subsection we illustrate the case of time-dependent perturbations.

### 2.3. The Augmented Phase-Space Method

To cast time-dependent perturbations within the framework of the exact response theory illustrated above, one can remove the time dependence introducing auxiliary variables, enlarging the phase space as follows. Given a generic dynamical system $\dot{\Gamma }=G\left(\Gamma \right)$ suppose we have an additive perturbation written in the form

(14) $$\dot{\Gamma }=G\left(\Gamma ,t\right)=G_{0}\left(\Gamma \right)+Fw\left(t\right),$$

where *G* and $G_{0}$ stand for perturbed and unperturbed vector fields, respectively. Since any perturbation can be interpreted as periodic with period *T*, such that *T* is enough larger than the observation scale time, we can rewrite any perturbation as a Fourier series:

(15) $$Fw\left(t\right)=\sum_{n=-\infty }^{+\infty }\alpha_{n}e^{i\beta_{n}t}=\sum_{n=-\infty }^{+\infty }\alpha_{n}w_{n}\left(\theta ,\phi \right)=w\left(\theta ,\phi \right),$$

where $\beta_{n}=2\pi n/T$ are the Fourier frequencies. In recent work [18], an analogous strategy has been carried out to handle stochastic perturbations, making use of the Karhunen–Loève expansion for Wiener and other stochastic processes. In practice, the main difference between the stochastic and deterministic perturbations is that the coefficients $\alpha_{n}$ are random variables in the first case and are fixed numbers in the second. Therefore, the two cases can be easily treated in parallel. In both cases, one may assume that the perturbation is periodic with a period much larger than any physically relevant time [2]. Then, this periodicity suggests the use of a harmonic oscillator in the space $M_{\theta \phi }$ to remove the time variable from the relevant vector field:

(16) $$\left\{\begin{array}{c} \dot{\theta }=\phi \\ \dot{\phi }=-\omega^{2}\theta . \end{array}\right.$$

The dynamics now lives in the augmented phase space $\tilde{M}:=M_{\Gamma }\times M_{\theta \phi }$ whose phase-space variable is $\tilde{\Gamma }=\left(\Gamma ,\theta ,\phi \right)$. Substituting (15) in (14), and explicitly writing the components of the vector field, we obtain the following:

(17) $$\dot{\tilde{\Gamma }}\left(t\right)=\tilde{G}\left(\Gamma ,\theta ,\phi \right)=\left(\begin{array}{c} G_{1}\left(\Gamma \right)+\sum_{n=-\infty }^{+\infty }\alpha_{n1}w_{n}\left(\theta ,\phi \right)\\ G_{2}\left(\Gamma \right)+\sum_{n=-\infty }^{+\infty }\alpha_{n2}w_{n}\left(\theta ,\phi \right)\\ \vdots \\ G_{N}\left(\Gamma \right)+\sum_{n=-\infty }^{+\infty }\alpha_{nN}w_{n}\left(\theta ,\phi \right)\\ \phi \\ -\omega^{2}\theta \end{array}\right)$$

where the angular variables obey Equation (16). In this way, the exact response theory can be applied, up to some adaptations to the current time-delayed case. In the augmented space, the phase-space variation rate $\tilde{\Lambda }=div\;\tilde{G}$ equals the unperturbed rate due to the null contribution of the harmonic oscillator dynamics:

(18) $$\tilde{\Lambda }\left(\tilde{\Gamma }\right)=\Lambda \left(\Gamma \right).$$

Given $f_{0}\left(\Gamma \right)$ and $g_{0}\left(\theta ,\phi \right)$ probability distributions over M and $M_{\theta \phi }$, respectively, the unperturbed distribution over the augmented phase space $d\tilde{\mu }$ on $\tilde{M}$ can be given by the probability density

(19) $${\tilde{f}}_{0}\left(\tilde{\Gamma },\theta ,\phi \right)=f_{0}\left(\Gamma \right)g_{0}\left(\theta ,\phi \right),$$

i.e., factorized in terms of the two independent distributions. Then, the dissipation function ${\tilde{\Omega }}^{{\tilde{f}}_{0}}$ for the augmented phase space takes the form [18]

(20) $$\begin{array}{cc} {\tilde{\Omega }}^{{\tilde{f}}_{0}}\left(\Gamma ,\theta ,\phi \right) & =-\tilde{\Lambda }\left(\Gamma ,\theta ,\phi \right)-\frac{d}{dt}\ln{\tilde{f}}_{0}\left(\Gamma ,\theta ,\phi \right)\\ \; & =-\frac{1}{{\tilde{f}}_{0}\left(\Gamma ,\theta ,\phi \right)}\nabla {\tilde{f}}_{0}\left(\Gamma ,\theta ,\phi \right)\cdot \tilde{G}\left(\Gamma ,\theta ,\phi \right)\\ \; & =\Omega^{f_{0}}\left(\Gamma \right)-\frac{1}{f_{0}\left(\Gamma \right)}\sum_{n_{-}\infty }^{+\infty }w_{n}\left(\theta ,\phi \right)\sum_{k=1}^{N}\alpha_{nk}\frac{\partial f_{0}}{\partial \Gamma_{k}}\left(\Gamma \right)\\ \; & \;-\frac{1}{g_{0}\left(\theta ,\phi \right)}\left(\phi \frac{\partial g_{0}}{\partial \theta }\left(\theta ,\phi \right)-\theta \frac{\partial g_{0}}{\partial \phi }\left(\theta ,\phi \right)\right) \end{array}$$

and the exact response for a generic observable O reads as

(21) $$\begin{array}{cc} E_{{\tilde{f}}_{t}}\left[O\right] & =E_{{\tilde{f}}_{0}}\left[O\right]+\int_{0}^{t}E_{{\tilde{f}}_{0}}\left[{\tilde{\Omega }}^{{\tilde{f}}_{0}}\left(\tilde{O}\circ {\tilde{S}}^{s}\right)\right]ds\\ \; & =E_{f_{0}}\left[O\right]+\int_{0}^{t}E_{{\tilde{f}}_{0}}\left[{\tilde{\Omega }}^{{\tilde{f}}_{0}}\left(\tilde{O}\circ {\tilde{S}}^{s}\right)\right]ds, \end{array}$$

where the first term in the right-hand side of the formula is equal to the average of the observable with respect to the unperturbed distribution, while the second expectation is evaluated over the extended phase space $M_{\theta \phi }$. More precisely,

(22) $$E_{{\tilde{f}}_{0}}\left[{\tilde{\Omega }}^{{\tilde{f}}_{0}}\left(\tilde{O}\circ {\tilde{S}}^{s}\right)\right]=\int_{M_{\theta \phi }}g_{0}\left(\theta ,\phi \right)d\theta d\phi \int_{M}{\tilde{\Omega }}^{{\tilde{f}}_{0}}\left(\Gamma ,\theta ,\phi \right)O\left(S^{s}\left(\Gamma \right)\right)f_{0}\left(\Gamma \right)d\Gamma ,$$

where, being that the observable O is only dependent on Γ, the embedding in the extended phase space $\tilde{\Gamma }$ does not change its values and one can state that $\tilde{O}\left({\tilde{S}}^{s}\left(\tilde{\Gamma }\right)\right)=O\left(S^{s}\left(\Gamma \right)\right)$, where $S^{s}$ expresses the perturbed evolution in the original phase space M, while ${\tilde{S}}^{s}$ expresses that in the augmented space $\tilde{M}$. The extra term takes into account the evolution of the time-dependent perturbation in the integral in $M_{\theta \phi }$, whose action on $w_{n}$ is an average with respect to $g_{0}\left(\theta ,\phi \right)$.

The choice of $g_{0}$ is independent of the dynamics and thus a convenient choice of distribution is the uniform one, since all derivatives vanish to 0 and ergodic consistency is not violated, as pointed out in the relevant work [6]; further details on the effect of a peaked distribution on specific initial conditions are given in Appendix B.

## 3. Delay Equation

We now turn to delay equations, illustrating with several examples how the exact response theory can be applied. We consider the following paradigmatic equation:

(23) $$\dot{v}\left(t\right)=-\int_{0}^{t}g\left(t-\zeta \right)v\left(\zeta \right)d\zeta =-\left(g*v\right)\left(t\right),$$

where *g* is the memory kernel. In this class of equations, the current rate of velocity change, $\dot{v}\left(t\right)$, depends not only on the current velocity *v*, but on the entire history of the system. However, the specific examples we consider only serve as illustrations of the applicability of the exact response theory. Whether the delay equations are solved analytically or numerically and the form of their solutions does not concern our work. Also, the symbol *v* is commonly associated with a velocity, but it may represent any quantity of interest.

It is reasonable to assume *v* and *g* Laplace-transformable on [0,+∞) as both the state variable and the convolution kernel are typically locally integrable. Thus we prescribe the following initial conditions for the dynamics (23), $v\left(0\right)=v_{0}$, $v^{\prime }\left(0\right)=0$, and introduce

(24) $$V\left(s\right)=L\left\{v\right\}\left(s\right)=\int_{0}^{+\infty }e^{-st}v\left(t\right)dt,\;G\left(s\right)=L\left\{g\right\}\left(s\right)=\int_{0}^{+\infty }e^{-st}g\left(t\right)dt,$$

where *s* denotes the Laplace domain variable. In order to recast the original differential equation into an algebraic form, we make use of the Laplace transform property for convolutions, obtaining

(25) $$v\left(t\right)=L^{-1}\left\{V\right\}\left(t\right)=L^{-1}\left\{\frac{v_{0}}{s-G(s)}\right\}\left(t\right).$$

For a vast set of phenomena, tempered kernels of the form

(26) $$g\left(t\right)=\frac{at^{\alpha -1}e^{-bt}}{\Gamma (\alpha )}$$

with $\alpha \in R^{+}$ are employed to describe anomalous transport scenarios like fractional diffusion [32,33,34] whenever α describes fractional dynamics, otherwise they can describe non-trivial memory effects. In return, the inverse Laplace transform of V(s) typically involves Prabhakar-type Mittag–Leffler [35] functions such as

(27) $$E_{\mu ,\nu }^{\gamma }\left(z\right)=\sum_{k=0}^{+\infty }\frac{z^{k}{\left(\gamma \right)}_{k}}{k!\Gamma (\mu k+\nu )}$$

of parameters μ, ν, and γ, or confluent hypergeometric functions [36,37]

(28) F11(a;b;z)=∑k=0+∞a(k)zkb(k)k!.

For this family of memory kernels, like for many others, solutions can be obtained with well-known techniques. However, we will only use them as illustrations of the applicability of the TTCF formalism, based on the extended phase space and dissipation function Ω. The purpose of the present paper is indeed to show how the response of dynamics expressed by delay equations can be approached within that formalism, that also provides techniques for the quantification of uncertainties. In the following we consider three main functional forms of g(t), the Erlang-type kernel, a particular kind of tempered kernel where parameter α in (26) is a positive integer, the exponential case, and the stepwise continuous function, through which one can arbitrarily closely approximate memory kernels of a vast class of forms.

### 3.1. Exponentially Decreasing Memory Kernel

The Erlang-type kernel with α=1 simply reduces to $g\left(t\right)=ae^{-bt}$, whose physical meaning is also known and used for modeling fast decaying memory, as for some viscoelasticity models [38] and brownian motion with memory effects. Substituting this functional form into (23), we obtain

(29) $$\ddot{v}\left(t\right)+b\dot{v}\left(t\right)+av\left(t\right)=0,$$

where the exponentially decaying memory allows the system to be recasted as a damped harmonic oscillator. Being that the system is autonomous, the exact response formalism can be directly applied by introducing auxiliary variables x(t):=v(t), $z\left(t\right):=\dot{x}\left(t\right)$:

(30) $$\dot{\Gamma }\left(t\right)=G\left(\Gamma \right)=\left(\begin{array}{c} z(t)\\ -ax(t)-bz(t) \end{array}\right),$$

with Γ=(x,z)∈M. The flow associated to the current dynamical system reads

(31) $$S^{t}\left(\begin{array}{c} x_{0}\\ z_{0} \end{array}\right)=\left(\begin{array}{c} x_{0}e^{-bt/2}\left(\cos\left(\omega t\right)+\frac{b}{2\omega }\sin\left(\omega t\right)\right)\\ -x_{0}\frac{a}{\omega }e^{-bt/2}\left(\sin(\omega t)\right) \end{array}\right),$$

where $\omega =\sqrt{4a-b^{2}}/2$ is the system’s natural oscillation frequency. Initial conditions are set to $x\left(0\right)=x_{0}$ and z(0)=0, consistent with the original system (23).

For the sake of illustration, we now choose a bivariate Gaussian distribution as the initial probability density:

(32) $$f_{0}\left(x,z\right)=\frac{1}{2\pi \sigma_{x}\sigma_{z}}\exp\left(-\frac{{\left(x-\mu_{x}\right)}^{2}}{2\sigma_{x}^{2}}-\frac{{\left(z-\mu_{z}\right)}^{2}}{2\sigma_{z}^{2}}\right).$$

Since the phase-space variation rate $\Lambda \left(\Gamma \right)=\nabla_{\Gamma }\cdot G\left(\Gamma \right)=-b$, the dissipation function reads as

(33) $$\Omega^{f_{0}}=-\Lambda \left(\Gamma \right)-\nabla_{\Gamma }\ln f_{0}\left(\Gamma \right)\cdot G\left(\Gamma \right)=z\frac{\left(x-\mu_{x}\right)}{\sigma_{x}^{2}}-\left(ax+bz\right)\frac{\left(z-\mu_{z}\right)}{\sigma_{z}^{2}}+b.$$

Let us now focus on the evolution of some physical observables. Taking O=x, since that the initial expectation $E_{f_{0}}\left[x\right]=\mu_{x}$, the correlation function yields

(34) $$\begin{array}{cc} \int_{0}^{t} & E_{f_{0}}\left[\Omega^{f_{0}}\left(x\circ S^{s}\right)\right]ds\\ \; & =\int_{0}^{t}\int_{M}\left(z\frac{\left(x-\mu_{x}\right)}{\sigma_{x}^{2}}-\left(ax+bz\right)\frac{\left(z-\mu_{z}\right)}{\sigma_{z}^{2}}+b\right)\left(\cos\left(\omega s\right)+\frac{b}{2\omega }\sin\left(\omega s\right)\right)x_{0}e^{-bs/2}\\ \; & \;\times \frac{1}{2\pi \sigma_{x}\sigma_{z}}\exp\left(-\frac{{\left(x-\mu_{x}\right)}^{2}}{2\sigma_{x}^{2}}-\frac{{\left(z-\mu_{z}\right)}^{2}}{2\sigma_{z}^{2}}\right)dxdzds\\ \; & =\frac{\mu_{z}}{2\pi \sigma_{x}\sigma_{z}}e^{-bt/2}\left(\frac{b}{2\omega }\sin\left(\omega t\right)-\cos\left(\omega t\right)\right) \end{array}$$

so that

(35) $$\begin{array}{cc} E_{f_{t}}\left[x\right] & =E_{f_{0}}\left[x\right]+\int_{0}^{t}E_{f_{0}}\left[\Omega^{f_{0}}\left(x\circ S^{s}\right)\right]ds\\ \; & =\mu_{x}+\frac{\mu_{z}}{2\pi \sigma_{x}\sigma_{z}}e^{-bt/2}\left(\frac{b}{2\omega }\sin\left(\omega t\right)-\cos\left(\omega t\right)\right). \end{array}$$

If we take $O=x^{2}$, we get that $E_{f_{0}}\left[x^{2}\right]=\sigma_{x}^{2}+\mu_{x}^{2}$ and

(36) $$\begin{array}{cc} \int_{0}^{t} & E_{f_{0}}\left[\Omega^{f_{0}}\left(x^{2}\circ S^{s}\right)\right]ds\\ \; & =\int_{0}^{t}\int_{M}\left(z\frac{\left(x-\mu_{x}\right)}{\sigma_{x}^{2}}-\left(ax+bz\right)\frac{\left(z-\mu_{z}\right)}{\sigma_{z}^{2}}+b\right)\\ \; & \;\times \left(\cos^{2}\left(\omega t\right)+\frac{b^{2}}{4\omega^{2}}\sin^{2}\left(\omega t\right)+\frac{b}{\omega }\cos\left(\omega t\right)\sin\left(\omega t\right)\right)x_{0}^{2}e^{-s}\\ \; & \;\times \frac{1}{2\pi \sigma_{x}\sigma_{z}}\exp\left(-\frac{{\left(x-\mu_{x}\right)}^{2}}{2\sigma_{x}^{2}}-\frac{{\left(z-\mu_{z}\right)}^{2}}{2\sigma_{z}^{2}}\right)dxdzds\\ \; & =\mu_{x}\mu_{z}\left(\frac{a+b^{2}}{ab}-e^{-bt}\left(\frac{a}{b\omega^{2}}+\frac{b(3a-b^{2})}{4a\omega^{2}}\cos\left(2\omega t\right)-\frac{a-b^{2}}{2a\omega }\sin\left(2\omega t\right)\right)\right), \end{array}$$

so that

(37) $$\begin{array}{cc} E_{f_{t}}\left[x^{2}\right] & =E_{f_{0}}\left[x^{2}\right]+\int_{0}^{t}E_{f_{0}}\left[\Omega^{f_{0}}\left(x^{2}\circ S^{s}\right)\right]ds\\ \; & =\sigma_{x}^{2}+\mu_{x}^{2}+\mu_{x}\mu_{z}\left(\frac{a+b^{2}}{ab}-e^{-bt}\left(\frac{a}{b\omega^{2}}+\frac{b(3a-b^{2})}{4a\omega^{2}}\cos\left(2\omega t\right)-\frac{a-b^{2}}{2a\omega }\sin\left(2\omega t\right)\right)\right). \end{array}$$

With the just computed two moments, we can now estimate the evolution of the uncertainty of the observable *x*, if it was initially given by the bivariate Gaussian (32).

Since the expected value for the observable O=z is simply $E_{f_{0}}\left[z\right]=\mu_{z}$, we have that

(38) $$\begin{array}{cc} \int_{0}^{t}E_{f_{0}} & [\Omega^{f_{0}}\left(z\circ S^{s}\right)]ds\\ \; & =\int_{0}^{t}\int_{M}\left(z\frac{\left(x-\mu_{x}\right)}{\sigma_{x}^{2}}-\left(ax+bz\right)\frac{\left(z-\mu_{z}\right)}{\sigma_{z}^{2}}+b\right)\left(-\frac{a}{\omega }\sin\left(\omega t\right)\right)x_{0}e^{-bs/2}\\ \; & \;\times \frac{1}{2\pi \sigma_{x}\sigma_{z}}\exp\left(-\frac{{\left(x-\mu_{x}\right)}^{2}}{2\sigma_{x}^{2}}-\frac{{\left(z-\mu_{z}\right)}^{2}}{2\sigma_{z}^{2}}\right)dxdzds\\ \; & =\frac{\mu_{z}}{2\pi \sigma_{x}\sigma_{z}}\left(-1+e^{-bt/2}\left(\cos\left(\omega t\right)+\frac{b}{2\omega }\sin\left(\omega t\right)\right)\right), \end{array}$$

hence finally we obtain

(39) $$\begin{array}{cc} E_{f_{t}}\left[z\right] & =E_{f_{0}}\left[z\right]+\int_{0}^{t}E_{f_{0}}\left[\Omega^{f_{0}}\left(z\circ S^{s}\right)\right]ds\\ \; & =\mu_{z}+\frac{\mu_{z}}{2\pi \sigma_{x}\sigma_{z}}\left(-1+e^{-bt/2}\left(\cos\left(\omega t\right)+\frac{b}{2\omega }\sin\left(\omega t\right)\right)\right). \end{array}$$

#### Comparison with Evolution in $\tilde{M}$

Let us now introduce a fictitious time-dependent perturbation w(t)=0 amplified by some factor *F*, with the purpose of representing the same system treated above within the extended theory, for time-dependent perturbations. The dynamical system now takes the form

(40) $$\dot{\Gamma }\left(t\right)=G\left(\Gamma \right)=\left(\begin{array}{c} z(t)\\ -ax(t)-bz(t)+Fw(t) \end{array}\right).$$

and, following our approach, it is first re-written over the extended phase space $\tilde{M}=M\times M_{\theta \phi }$ as

(41) $$\dot{\tilde{\Gamma }}\left(t\right)=\tilde{G}\left(\tilde{\Gamma }\right)=\left(\begin{array}{c} z(t)\\ -ax(t)-bz(t)+w(\theta ,\phi )\\ \phi (t)\\ -\omega^{2}\theta \left(t\right) \end{array}\right).$$

The corresponding evolution in $\tilde{M}$ follows

(42) $${\tilde{S}}^{t}\left(\begin{array}{c} x_{0}\\ z_{0}\\ \theta_{0}\\ \phi_{0} \end{array}\right)=\left(\begin{array}{c} x_{0}e^{-bt/2}\left(\cos\left(\omega t\right)+\frac{b}{2\omega }\sin\left(\omega t\right)\right)\\ -x_{0}e^{-bt/2}\frac{a}{\omega }\sin\left(\omega t\right)\\ \theta_{0}\cos\left(\omega t\right)+\frac{\phi_{0}}{\omega }\sin\left(\omega t\right)\\ \phi_{0}\cos\left(\omega t\right)-\omega \theta_{0}\sin\left(\omega t\right) \end{array}\right).$$

The bivariate Gaussian (32), over the original phase space M, still is the unperturbed probability density. Then, we define the initial distribution in the augmented phase space as

(43) $$\tilde{f_{0}}\left(\Gamma ,\theta ,\phi \right)=f_{0}\left(\Gamma \right)g_{0}\left(\theta ,\phi \right),$$

choosing the uniform distribution over $M_{\theta \phi }$ with $F=\sqrt{\theta_{0}^{2}+\phi_{0}^{2}}$, so that $g_{0}\left(\theta ,\phi \right)=1/2\pi \rho$. In principle, this expression allows a variety of initial values for the time-dependent perturbation, unlike the usual expression that has a fixed initial value. Although irrelevant in the present case, because the perturbation vanishes, this is useful in general, as it allows one to also study the consequences of the distribution of the initial perturbation values. These, like any other physical quantity, are typically affected by errors or uncertainties.

Given that $\tilde{\Lambda }\left(\tilde{\Gamma }\right)=\Lambda \left(\Gamma \right)=-b$, the dissipation function over the augmented phase space now reads as

(44) $$\begin{array}{cc} {\tilde{\Omega }}^{{\tilde{f}}_{0}}\left(\Gamma ,\theta ,\phi \right) & =-\tilde{\Lambda }\left(\tilde{\Gamma }\right)-\frac{1}{{\tilde{f}}_{0}\left(\Gamma ,\theta ,\phi \right)}\nabla {\tilde{f}}_{0}\left(\Gamma ,\theta ,\phi \right)\cdot \tilde{G}\left(\Gamma ,\theta ,\phi \right)\\ \; & =-\Lambda \left(\Gamma \right)-\frac{1}{f_{0}\left(\Gamma \right)}\nabla f_{0}\left(\Gamma \right)\cdot G\left(\Gamma \right)\\ \; & =\Omega^{f_{0}}\left(\Gamma \right), \end{array}$$

as required, since Fw(t)=w(θ,ϕ)=0. Similarly, by choosing a generic observable O=O(Γ) and evaluating the response to the perturbation, we get

(45) $$E_{{\tilde{f}}_{t}}\left[O\right]=E_{{\tilde{f}}_{0}}\left[O\right]+\int_{0}^{t}E_{{\tilde{f}}_{0}}\left[{\tilde{\Omega }}^{{\tilde{f}}_{0}}\left(\tilde{O}\circ {\tilde{S}}^{s}\right)\right]ds$$

but since ${\tilde{\Omega }}^{{\tilde{f}}_{0}}\left(\Gamma ,\theta ,\phi \right)=\Omega^{f_{0}}\left(\Gamma \right)$, it is clear that the initial expectation value in $\tilde{M}$

(46) $$\begin{array}{cc} E_{{\tilde{f}}_{0}}\left[O\right] & =\int_{\tilde{M}}O{\tilde{f}}_{0}\left(\tilde{\Gamma }\right)d\tilde{\Gamma }=\int_{M\times M_{\theta \phi }}Of_{0}\left(\Gamma \right)g_{0}\left(\theta ,\phi \right)d\Gamma d\theta d\phi \\ \; & =\int_{M_{\theta \phi }}g_{0}\left(\theta ,\phi \right)d\theta d\phi \int_{M}Of_{0}\left(\Gamma \right)d\Gamma \\ & =E_{f_{0}}\left[O\right] \end{array}$$

equals its value in M, as required, and the correlation function in $\tilde{M}$

(47) $$\begin{array}{cc} E_{{\tilde{f}}_{0}}\left[{\tilde{\Omega }}^{{\tilde{f}}_{0}}\left(\tilde{O}\circ {\tilde{S}}^{s}\right)\right] & =\int_{\tilde{M}}{\tilde{\Omega }}^{{\tilde{f}}_{0}}\left(\tilde{O}\circ {\tilde{S}}^{s}\right){\tilde{f}}_{0}\left(\tilde{\Gamma }\right)d\tilde{\Gamma }\\ & =\int_{M\times M_{\theta \phi }}\Omega^{f_{0}}\left(O\circ S^{s}\right)f_{0}\left(\Gamma \right)g_{0}\left(\theta ,\phi \right)d\Gamma d\theta d\phi \\ \; & =\int_{M_{\theta \phi }}g_{0}\left(\theta ,\phi \right)d\theta d\phi \int_{M}\Omega^{f_{0}}\left(O\circ S^{s}\right)f_{0}\left(\Gamma \right)d\Gamma \\ \; & =E_{f_{0}}\left[\Omega^{f_{0}}\left(O\circ S^{s}\right)\right]. \end{array}$$

does too. This simple example illustrates how the calculations in the augmented phase space reproduce those in the original phase space, as should be. However, in the augmented space, one can additionally treat the possible, and in practice unavoidable, statistic of the time-dependent perturbations.

### 3.2. Second Order Erlang-Type Kernel

We now consider kernel (26) with the choice of α=2, so that $g\left(t\right)=ate^{-bt}$, with a,b>0. This way the dynamics prescribed by (23) reads as

(48) $$\dot{v}\left(t\right)=-\int_{0}^{t}g\left(t-s\right)v\left(s\right)ds=-\int_{0}^{t}a\left(t-s\right)e^{-b(t-s)}v\left(s\right)ds,$$

which can be recast in a three-dimensional ODE system of the form

(49) $$\left\{\begin{array}{c} \dot{x}\left(t\right)=y\left(t\right)\\ \dot{y}\left(t\right)=-by\left(t\right)+z\left(t\right)\\ \dot{z}\left(t\right)=-ax\left(t\right)-bz\left(t\right) \end{array}\right.$$

upon introducing the following auxiliary variables:

(50) $$x\left(t\right):=v\left(t\right),\;y\left(t\right):=\dot{v}\left(t\right)=-\int_{0}^{t}a\left(t-s\right)e^{-b(t-s)}v\left(s\right)ds,\;z\left(t\right):=-\int_{0}^{t}ae^{-b(t-s)}v\left(s\right)ds.$$

In vector formulation,

(51) $$\dot{\Gamma }\left(t\right)=\left(\begin{array}{c} \dot{x}\\ \dot{y}\\ \dot{z} \end{array}\right)=\left(\begin{array}{c} y\\ -by+z\\ -ax-bz \end{array}\right)=G\left(\Gamma \right),$$

it is possible to compute the phase-space contraction rate Λ(Γ)=∇·G(Γ)=−2b and the flow operator

(52) Ssx0y0z0=x0∑k=0+∞akt3k(3k)!F11(2k;3k+1;−bt)x0a∑k=0∞akt3k+2(3k+2)!F11(2k+2;3k+3;−bt)x0∑k=0∞akt3k+1(3k+1)!F11(2k+2;3k+2;−bt)+bakt3k(3k)!F11(2k;3k+1;−bt)

with compatible initial conditions $x\left(0\right)=v_{0}$, y(0)=0, and z(0)=0. Assuming a multivariate Gaussian distribution as the equilibrium pdf,

(53) $$f_{0}\left(x,y,z\right)=\frac{1}{{\left(2\pi \right)}^{3/2}\sigma_{x}\sigma_{y}\sigma_{z}}\exp\left(-\frac{{\left(x-\mu_{x}\right)}^{2}}{2\sigma_{x}^{2}}-\frac{{\left(y-\mu_{y}\right)}^{2}}{2\sigma_{y}^{2}}-\frac{{\left(z-\mu_{z}\right)}^{2}}{2\sigma_{z}^{2}}\right),$$

the dissipation function is as follows:

(54) $$\Omega^{f_{0}}=-\Lambda \left(\Gamma \right)-\nabla_{\Gamma }\ln f_{0}\left(\Gamma \right)=-2b-y\frac{\left(x-\mu_{x}\right)}{\sigma_{x}^{2}}-\left(-by+z\right)\frac{\left(y-\mu_{y}\right)}{\sigma_{y}^{2}}+\left(ax+bz\right)\frac{\left(z-\mu_{z}\right)}{\sigma_{z}^{2}}.$$

Considering now the evolution of observable O=x, that describes the system’s velocity, being the expectation $E_{f_{0}}\left[x\right]=\mu_{x}$, the correlation function yields

(55) Ef0[Ωf0(x∘Ss)]=1(2π)3/2σxσyσz∫M−2b−y(x−μx)σx2−(−by+z)(y−μy)σy2+(ax+bz)(z−μz)σz2×x∑k=0+∞aks3k(3k)!F11(2k;3k+1;−bs)exp−(x−μx)22σx2−(y−μy)22σy2−(z−μz)22σz2dxdydz.

Considering instead $O=x^{2}$, related to the energy of the system, we first recall that $E_{f_{0}}\left[x^{2}\right]=\mu_{x}^{2}+\sigma_{x}^{2}$ and express the correlation function as

(56) Ef0[Ωf0(x2∘Ss)]=1(2π)3/2σxσyσz∫M−2b−y(x−μx)σx2−(−by+z)(y−μy)σy2+(ax+bz)(z−μz)σz2×x∑k=0+∞aks3k(3k)!F11(2k;3k+1;−bs)2exp−(x−μx)22σx2−(y−μy)22σy2−(z−μz)22σz2dxdydz.

Hence, finally, when considering O=y, i.e., the system’s acceleration, we first recall $E_{f_{0}}\left[y\right]=\mu_{y}$, and the correlation function yields

(57) Ef0[Ωf0(y∘Ss)]=1(2π)3/2σxσyσz∫M−2b−y(x−μx)σx2−(−by+z)(y−μy)σy2+(ax+bz)(z−μz)σz2×ax∑k=0∞aks3k+2(3k+2)!F11(2k+2;3k+3;−bs)×exp−(x−μx)22σx2−(y−μy)22σy2−(z−μz)22σz2dxdydz.

Regardless of the specific choice of the observable, adopting the response formula in (13) is sufficient to connect the correlation function with the response of the considered physical variable at time *t*.

### 3.3. Simple Function Memory Kernel

Another interesting example of delay equations can be constructed in terms of linear combinations of step functions, also known as simple functions. These can, in fact, approximate as accurately as needed all integrable functions. Let us begin with a free particle subjected to a retarded friction γ made of *N* steps, each acting for a time τ:

(58) $$\gamma \left(t\right)=\sum_{i=1}^{N}\gamma_{i}\chi_{\left((i-1)\tau ,i\tau \right]}\left(t\right)=\left\{\begin{array}{cc} \gamma_{1}\; & t\in (0,\tau ]\\ \gamma_{2}\; & t\in (\tau ,2\tau ]\\ \vdots \\ \gamma_{N} & t\in \left((N-1)\tau ,N\tau \right]\\ 0 & t\notin (0,N\tau ] \end{array}\right.,\;\gamma_{i}\in R,$$

assuming that no memory is present up to t=0. By plugging this memory kernel inside the integro-differential Equation (23) and noticing that the term −γ(0)v(t)=0 under our assumptions, a convenient reformulation of (58) yields

(59) $$\begin{array}{cc} \ddot{v}\left(t\right) & =-\int_{0}^{t}\frac{\partial }{\partial t}\left(\sum_{k=1}^{N}\gamma_{k}\left[H(t-(k-1)\tau )-H(t-k\tau )\right]\right)v\left(s\right)ds\\ \; & =-\int_{0}^{t}\left(\gamma_{1}\delta \left(t-s\right)+\sum_{k=2}^{N}\left(\gamma_{k}-\gamma_{k-1}\right)\delta \left(t-s-(k-1)\tau \right)-\gamma_{N}\delta \left(t-s-N\tau \right)\right)v\left(s\right)ds \end{array}$$

which can be written as

(60) $$\ddot{v}\left(t\right)+\gamma_{1}v\left(t\right)=-\sum_{k=2}^{N}\left(\gamma_{k}-\gamma_{k-1}\right)v\left(t-(k-1)\tau \right)H\left(t-(k-1)\tau \right)+\gamma_{N}v\left(t-N\tau \right)H\left(t-N\tau \right),$$

that represents a harmonic oscillator subjected to a cumulative time-delayed forcing that follows specific rules. For t∈(0,τ], no forcing is present, while as time grows above τ, additional memory effects play the role of perturbations to the original dynamics. As time passes, new contributions to the dynamics appear due to past memory terms, whose intensity depends on the variation rates $\Delta \gamma_{k}=\gamma_{k}-\gamma_{k-1}$. For t∈(0,τ], we have

(61) $$\ddot{v}\left(t\right)+\gamma_{1}v\left(t\right)=0,$$

which is the equation of a harmonic oscillator in M in which the role of the “position” of the oscillator is given by the velocity and whose solution can be written as $v\left(t\right)=v_{0}\cos\left(\sqrt{\gamma_{1}}t\right)$, if the initial conditions are assumed to be $v\left(0\right)=v_{0}$ and $\dot{v}\left(0\right)=0$. For t∈(τ,2τ], we have

(62) $$\ddot{v}\left(t\right)+\gamma_{1}v\left(t\right)=-\left(\gamma_{2}-\gamma_{1}\right)v\left(t-\tau \right),$$

where v(t−τ) is the solution coming from (61), delayed in time by a factor τ. In other words the system at step 2 becomes

(63) $$\ddot{v}\left(t\right)+\gamma_{1}v\left(t\right)=v_{0}\left(\gamma_{1}-\gamma_{2}\right)\cos\left(\sqrt{\gamma_{1}}\left(t-\tau \right)\right),$$

an ordinary delayed differential equation, whose delay is included in the forcing term; for more details for an alternative proof see Appendix A. As time grows, additional delays arise to continue the solution of the previous step in a differentiable fashion, which leads to

(64) $$\begin{array}{cc} \ddot{v}\left(t\right)+\gamma_{1}v\left(t\right) & =v_{0}\left(\gamma_{1}-\gamma_{2}\right)\left(\cos\left(\sqrt{\gamma_{1}}\tau \right)\cos\left(\sqrt{\gamma_{1}}t\right)+\sin\left(\sqrt{\gamma_{1}}\tau \right)\sin\left(\sqrt{\gamma_{1}}t\right)\right)\\ \; & =v_{0}\left(\gamma_{1}-\gamma_{2}\right)\left(A\left(\tau \right)\cos\left(\sqrt{\gamma_{1}}t\right)+B\left(\tau \right)\sin\left(\sqrt{\gamma_{1}}t\right)\right), \end{array}$$

where the delay is expressed by the driving terms $A\left(\tau \right)=\cos\left(\sqrt{\gamma }\tau \right)$ and $B\left(\tau \right)=\sin\left(\sqrt{\gamma }\tau \right)$. With the necessary caution, this process can be repeated to reach any time t>0.

For the applicability of the exact response formalism to this class of delayed systems, let us start from the first step where, for later convenience, we shall call x(t) our physical (velocity) variable and z(t) its time derivative. Letting $\gamma_{1}=\gamma$ for simplicity, we can write

(65) $$\left\{\begin{array}{c} \dot{x}\left(t\right)=z\left(t\right)\\ \dot{z}\left(t\right)=-\gamma x\left(t\right) \end{array}\right.\;or\;\;\;\dot{\Gamma }\left(t\right)=G\left(\Gamma \right)=\left(\begin{array}{c} G_{1}\left(\Gamma \right)\\ G_{2}\left(\Gamma \right) \end{array}\right).$$

The flow operator then reads as

(66) $$S^{s}\left(\begin{array}{c} x_{0}\\ z_{0} \end{array}\right)=\left(\begin{array}{c} x_{0}\cos\left(\sqrt{\gamma }t\right)\\ -\sqrt{\gamma }x_{0}\sin\left(\sqrt{\gamma }t\right) \end{array}\right),$$

having assumed $x\left(0\right)=x_{0}$ and z(0)=0 as the initial conditions, consistently with the original integro-differential equation. As the phase-space variation rate vanishes, Λ(Γ)=0, the dissipation function reads as

(67) $$\Omega^{f_{0}}\left(\Gamma \right)=-\frac{1}{f_{0}\left(x,z\right)}\nabla f_{0}\left(x,z\right)\cdot \left(\begin{array}{c} z\\ -\gamma x \end{array}\right)=\frac{\left(x-\mu_{x}\right)}{\sigma_{x}^{2}}z-\frac{\left(z-\mu_{z}\right)}{\sigma_{z}^{2}}\gamma x.$$

Since no memory effect is present, the time-independent exact response formula could be initially applied to the evolution of the average of the observable. However, it is better to use the form defined in the augmented phase space, that becomes necessary for times larger than τ. In the second step, t∈(τ,2τ], we take $\gamma_{2}=0$ and $\gamma_{1}=\gamma$, implying that memory lasts only for a time τ. The delayed system becomes

(68) $$\ddot{x}\left(t\right)+\gamma x\left(t\right)=x_{0}\gamma \cos\left(\sqrt{\gamma }\left(t-\tau \right)\right),$$

which can be cast in the form

(69) $$\left\{\begin{array}{c} \dot{x}\left(t\right)=z\left(t\right)\\ \dot{z}\left(t\right)=-\gamma x\left(t\right)+x_{0}\gamma \cos\left(\sqrt{\gamma }\left(t-\tau \right)\right)=-\gamma x\left(t\right)+Fw\left(t,\tau \right), \end{array}\right.$$

where the term Fw(t,τ), with $F=x_{0}\gamma$, accounts for a time-delayed forcing and is not present in the first step. To account for such forcing we move from the basic dynamics $\dot{\Gamma }\left(t\right)=G\left(\Gamma \right)$ to

(70) $$\dot{\tilde{\Gamma }}\left(t\right)=\left(\begin{array}{c} G_{1}\left(\Gamma \right)\\ G_{2}\left(\Gamma \right)+Fw\left(t\right)\\ \phi \\ -\omega^{2}\theta \end{array}\right).$$

This is done by introducing two auxiliary variables, θ and ϕ, that obey

(71) $$\left\{\begin{array}{c} \dot{\theta }=\phi \\ \dot{\phi }=-\omega^{2}\theta \end{array}\right.$$

in the corresponding extended phase space $M_{\theta \phi }$. This allows us to re-write the delay Fw(t) as a time-independent term in $\tilde{M}$:

(72) Fw(t)=A(τ)ϕ+B(τ)θ=w(θ,ϕ),

with (θ,ϕ)=(sin(ωt),cos(ωt)). In Equation (15), we now have a single frequency, which is $\sqrt{\gamma }$, and $\alpha_{n}=\delta_{n,1}$ where $\delta_{i,j}$ is the Kronecker symbol. Therefore, the dynamics of the system can now be approached as an autonomous dynamical system in $\tilde{M}$ given by the following equations:

(73) $$\dot{\tilde{\Gamma }}\left(t\right)=\tilde{G}\left(\Gamma ,\theta ,\phi \right)=\left(\begin{array}{c} G_{1}\left(\Gamma \right)\\ G_{2}\left(\Gamma \right)+Fw\left(\theta ,\phi \right)\\ \phi \\ -\gamma \theta \end{array}\right)=\left(\begin{array}{c} z\\ -\gamma x+A(\tau )\phi +B(\tau )\theta \\ \phi \\ -\gamma \theta . \end{array}\right).$$

Referring to the dynamics in (73), the flow operator, with generic initial conditions $\left(x_{\tau },z_{\tau },\theta_{\tau },\phi_{\tau }\right)$, can be expressed as

(74) $$\;{\tilde{S}}^{s}\left(\begin{array}{c} x_{\tau }\\ z_{\tau }\\ \theta_{\tau }\\ \phi_{\tau } \end{array}\right)=\left(\begin{array}{c} \left(A\left(\tau \right)x_{\tau }-\frac{B\left(\tau \right)K_{2}}{2\sqrt{\gamma }}-\frac{B\left(\tau \right)z_{\tau }}{\sqrt{\gamma }}-\frac{\left(t-\tau \right)}{2\sqrt{\gamma }}\left(B\left(\tau \right)K_{1}+A\left(\tau \right)K_{2}\right)\right)\cos\left(\sqrt{\gamma }t\right)\\ +\left(B\left(\tau \right)x_{\tau }+\frac{A\left(\tau \right)K_{2}}{2\sqrt{\gamma }}-\frac{A\left(\tau \right)z_{\tau }}{\sqrt{\gamma }}+\frac{\left(t-\tau \right)}{2\sqrt{\gamma }}\left(A\left(\tau \right)K_{1}-B\left(\tau \right)K_{2}\right)\right)\sin\left(\sqrt{\gamma }t\right)\\ \left(A\left(\tau \right)z_{\tau }-\frac{B\left(\tau \right)K_{1}}{2\sqrt{\gamma }}+B\left(\tau \right)x_{\tau }\sqrt{\gamma }+\frac{\left(t-\tau \right)}{2}\left(A\left(\tau \right)K_{1}-B\left(\tau \right)K_{2}\right)\right)\cos\left(\sqrt{\gamma }t\right)\\ +\left(B\left(\tau \right)z_{\tau }+\frac{A\left(\tau \right)K_{1}}{2\sqrt{\gamma }}-A\left(\tau \right)x_{\tau }\sqrt{\gamma }+\frac{\left(t-\tau \right)}{2}\left(A\left(\tau \right)K_{2}+B\left(\tau \right)K_{1}\right)\right)\sin\left(\sqrt{\gamma }t\right)\\ \left(A\left(\tau \right)\theta_{\tau }-\frac{B\left(\tau \right)\phi_{\tau }}{\sqrt{\gamma }}\right)\cos\left(\sqrt{\gamma }t\right)+\left(B\left(\tau \right)\theta_{\tau }+\frac{A\left(\tau \right)\phi_{\tau }}{\sqrt{\gamma }}\right)\sin\left(\sqrt{\gamma }t\right)\\ \left(B\left(\tau \right)\theta_{\tau }\sqrt{\gamma }+A\left(\tau \right)\phi_{\tau }\right)\cos\left(\sqrt{\gamma }t\right)+\left(-A\left(\tau \right)\theta_{\tau }\sqrt{\gamma }+B\left(\tau \right)\phi_{\tau }\right)\sin\left(\sqrt{\gamma }t\right) \end{array}\right)$$

where

(75) $$K_{1}=B\theta_{\tau }+A\phi_{\tau },\;K_{2}=\frac{B\phi_{\tau }}{\sqrt{\gamma }}-A\sqrt{\gamma }\theta_{\tau }.$$

As in the previous example, we take the initial probability density in the augmented phase space given by Equations (19) and (32) and $g_{0}\left(\theta ,\phi \right)=1/2\pi \rho$, with $\rho =\sqrt{\theta_{0}^{2}+\phi_{0}^{2}}$. This allows us to write the following:

(76) $$\begin{array}{cc} {\tilde{\Omega }}^{{\tilde{f}}_{0}}\left(\Gamma ,\theta ,\phi \right) & =-\tilde{\Lambda }\left(\Gamma ,\theta ,\phi \right)-\frac{d}{dt}\ln{\tilde{f}}_{0}\left(\Gamma ,\theta ,\phi \right)=-\frac{1}{{\tilde{f}}_{0}\left(\Gamma ,\theta ,\phi \right)}\nabla {\tilde{f}}_{0}\left(\Gamma ,\theta ,\phi \right)\cdot \tilde{G}\left(\Gamma ,\theta ,\phi \right)\\ \; & =\Omega^{f_{0}}\left(\Gamma \right)-\frac{1}{f_{0}\left(x,z\right)}\left[\frac{\partial f_{0}\left(x,z\right)}{\partial z}w\left(\theta ,\phi \right)\right]\\ \; & =\Omega^{f_{0}}\left(\Gamma \right)+\frac{\left(z-\mu_{z}\right)}{\sigma_{z}^{2}}\left(A(\tau )\phi +B(\tau )\theta \right). \end{array}$$

We note that this dissipation function equals the dissipation function for the original dynamics with an extra term concerning the contribution of the auxiliary dynamics, which is linear in both θ and ϕ. Even if the dynamics may look formal without memory, because the corresponding kernel is piecewise constant, some kind of dissipation arises because it still changes in time. The exact response formula now reads as

(77) $$\begin{array}{cc} E_{{\tilde{f}}_{t}}\left[O\right] & =E_{f_{0}}\left[O\right]+\int_{0}^{t}E_{{\tilde{f}}_{0}}\left[\left(O\circ {\tilde{S}}^{s}\right){\tilde{\Omega }}^{{\tilde{f}}_{0}}\right]ds\\ \; & =E_{f_{0}}\left[O\right]+\int_{0}^{t}\int_{M}\left(O\circ {\tilde{S}}^{s}\right)\Omega^{f_{0}}f_{0}\left(\Gamma \right)d\Gamma ds\\ \; & \;+\int_{\tau }^{t}\int_{\tilde{M}}\left(O\circ {\tilde{S}}^{s}\right)\left(A(\tau )\phi +B(\tau )\theta \right)\frac{\left(z-\mu_{z}\right)}{\sigma_{z}^{2}}{\tilde{f}}_{0}\left(\tilde{\Gamma }\right)d\tilde{\Gamma }ds\\ \; & =E_{f_{0}}\left[O\right]+\int_{0}^{\tau }\int_{M}\left(O\circ S^{s}\right)\Omega^{f_{0}}f_{0}\left(\Gamma \right)d\Gamma ds\\ \; & \;+\int_{\tau }^{t}\int_{M}\left(O\circ {\tilde{S}}^{s}\right)\Omega^{f_{0}}f_{0}\left(\Gamma \right)d\Gamma ds\\ \; & \;+\int_{\tau }^{t}\int_{\tilde{M}}\left(O\circ {\tilde{S}}^{s}\right)\left(A(\tau )\phi +B(\tau )\theta \right)\frac{\left(z-\mu_{z}\right)}{\sigma_{z}^{2}}{\tilde{f}}_{0}\left(\tilde{\Gamma }\right)d\tilde{\Gamma }ds. \end{array}$$

This equation shows that the exact response in $\tilde{M}$ is the one for the time-dependent perturbation with an additional term produced by the variables of the auxiliary space. That represents the possibility of considering the initial values of the time-dependent perturbation to be random and distributed according to some given law, while usually these initial values are fixed. Nevertheless, in some cases, the existing symmetries yield zero for this term. Consider, for instance, the observable O=x, since trivially $E_{f_{0}}\left[x\right]=\mu_{x}$, we can write

(78) $$\begin{array}{cc} \; & \int_{0}^{\tau }\int_{M}\left(x\circ S^{s}\right)\Omega^{f_{0}}f_{0}\left(\Gamma \right)d\Gamma ds=\frac{1}{2\pi \sigma_{x}\sigma_{z}}\int_{0}^{\tau }\int_{M}\left(x\cos(\sqrt{\gamma }s)\right)\left(\frac{\left(x-\mu_{x}\right)}{\sigma_{x}^{2}}z-\frac{\left(z-\mu_{z}\right)}{\sigma_{z}^{2}}\gamma x\right)\\ \; & \;\times \exp\left(-\frac{{\left(x-\mu_{x}\right)}^{2}}{2\sigma_{x}^{2}}-\frac{{\left(z-\mu_{z}\right)}^{2}}{2\sigma_{z}^{2}}\right)dxdzds=\frac{\mu_{z}}{\sqrt{\gamma }}\left(\sin\left(\sqrt{\gamma }t\right)-\sin\left(\sqrt{\gamma }\tau \right)\right)) \end{array}$$

and

(79) $$\begin{array}{cc} \; & \int_{\tau }^{t}\int_{M}\left(x\circ {\tilde{S}}^{s}\right)\Omega^{f_{0}}f_{0}\left(\Gamma \right)d\Gamma ds=\frac{1}{2\pi \sigma_{x}\sigma_{z}}\int_{\tau }^{t}\int_{M}[A\left(\tau \right)x-\frac{B\left(\tau \right)K_{2}}{2\sqrt{\gamma }}-\frac{B(\tau )z}{\sqrt{\gamma }}\\ \; & \;-\frac{\left(t-\tau \right)}{2\sqrt{\gamma }}\left(B\left(\tau \right)K_{1}+A\left(\tau \right)K_{2}\right))\cos\left(\sqrt{\gamma }s\right)+(B\left(\tau \right)x+\frac{A\left(\tau \right)K_{2}}{2\sqrt{\gamma }}-\frac{A(\tau )z}{\sqrt{\gamma }}\\ \; & \;+\frac{\left(t-\tau \right)}{2\sqrt{\gamma }}\left(A\left(\tau \right)K_{1}-B\left(\tau \right)K_{2}\right))\sin\left(\sqrt{\gamma }s\right)]\left(\frac{\left(x-\mu_{x}\right)}{\sigma_{x}^{2}}z-\frac{\left(z-\mu_{z}\right)}{\sigma_{z}^{2}}\gamma x\right)\\ \; & \;\times \exp\left(-\frac{{\left(x-\mu_{x}\right)}^{2}}{2\sigma_{x}^{2}}-\frac{{\left(z-\mu_{z}\right)}^{2}}{2\sigma_{z}^{2}}\right)dxdzds\\ \; & =\frac{1}{2\pi \sigma_{x}\sigma_{z}}\int_{\tau }^{t}\int_{M}[\left(A\left(\tau \right)\cos\left(\sqrt{\gamma }s\right)+B\left(\tau \right)\sin\left(\sqrt{\gamma }s\right)\right)x\\ \; & \;-\left(A\left(\tau \right)\sin\left(\sqrt{\gamma }s\right)+B\left(\tau \right)\cos\left(\sqrt{\gamma }s\right)\right)\frac{z}{\sqrt{\gamma }})]\left(\frac{\left(x-\mu_{x}\right)}{\sigma_{x}^{2}}z-\frac{\left(z-\mu_{z}\right)}{\sigma_{z}^{2}}\gamma x\right)\\ \; & \;\times \exp\left(-\frac{{\left(x-\mu_{x}\right)}^{2}}{2\sigma_{x}^{2}}-\frac{{\left(z-\mu_{z}\right)}^{2}}{2\sigma_{z}^{2}}\right)dxdzds, \end{array}$$

while

(80) $$\begin{array}{cc} \; & \int_{\tau }^{t}\int_{\tilde{M}}\left(x\circ {\tilde{S}}^{s}\right)\left(A(\tau )\phi +B(\tau )\theta \right)\frac{\left(z-\mu_{z}\right)}{\sigma_{z}^{2}}{\tilde{f}}_{0}\left(\tilde{\Gamma }\right)d\tilde{\Gamma }ds\\ \; & =\frac{1}{2\pi \rho }\int_{M_{\theta \phi }}\left(A(\tau )\phi +B(\tau )\theta \right)d\theta d\phi \int_{\tau }^{t}\frac{1}{2\pi \sigma_{x}\sigma_{z}}\int_{M}\frac{\left(z-\mu_{z}\right)}{\sigma_{z}^{2}}[A\left(\tau \right)x-\frac{B\left(\tau \right)K_{2}}{2\sqrt{\gamma }}\\ \; & \;-\frac{B(\tau )z}{\sqrt{\gamma }}-\frac{\left(t-\tau \right)}{2\sqrt{\gamma }}\left(B\left(\tau \right)K_{1}+A\left(\tau \right)K_{2}\right))\cos\left(\sqrt{\gamma }s\right)+(B\left(\tau \right)x+\frac{A\left(\tau \right)K_{2}}{2\sqrt{\gamma }}-\frac{A(\tau )z}{\sqrt{\gamma }}\\ \; & \;+\frac{\left(t-\tau \right)}{2\sqrt{\gamma }}\left(A\left(\tau \right)K_{1}-B\left(\tau \right)K_{2}\right))\sin\left(\sqrt{\gamma }s\right)]\exp\left(-\frac{{\left(x-\mu_{x}\right)}^{2}}{2\sigma_{x}^{2}}-\frac{{\left(z-\mu_{z}\right)}^{2}}{2\sigma_{z}^{2}}\right)dxdzds\\ \; & =\frac{1}{{\left(2\pi \right)}^{2}\rho \sigma_{x}\sigma_{z}}\int_{M_{\theta \phi }}\left(A(\tau )\phi +B(\tau )\theta \right)d\theta d\phi \int_{\tau }^{t}\int_{M}\frac{\left(z-\mu_{z}\right)}{\sigma_{z}^{2}}[\left(A\left(\tau \right)\cos\left(\sqrt{\gamma }s\right)+B\left(\tau \right)\sin\left(\sqrt{\gamma }s\right)\right)x\\ \; & \;-\left(A\left(\tau \right)\sin\left(\sqrt{\gamma }s\right)+B\left(\tau \right)\cos\left(\sqrt{\gamma }s\right)\right)\frac{z}{\sqrt{\gamma }})]\exp\left(-\frac{{\left(x-\mu_{x}\right)}^{2}}{2\sigma_{x}^{2}}-\frac{{\left(z-\mu_{z}\right)}^{2}}{2\sigma_{z}^{2}}\right)dxdzds. \end{array}$$

Considering instead $O=x^{2}$, since $E_{f_{0}}\left[x^{2}\right]=\mu_{x}^{2}+\sigma_{x}^{2}$, we can write

(81) $$\begin{array}{cc} \; & \int_{0}^{\tau }\int_{M}\left(x^{2}\circ S^{s}\right)\Omega^{f_{0}}f_{0}\left(\Gamma \right)d\Gamma ds=\frac{1}{2\pi \sigma_{x}\sigma_{z}}\int_{0}^{\tau }\int_{M}{\left(x\cos(\sqrt{\gamma }s)\right)}^{2}\left(\frac{\left(x-\mu_{x}\right)}{\sigma_{x}^{2}}z-\frac{\left(z-\mu_{z}\right)}{\sigma_{z}^{2}}\gamma x\right)\\ \; & \;\times \exp\left(-\frac{{\left(x-\mu_{x}\right)}^{2}}{2\sigma_{x}^{2}}-\frac{{\left(z-\mu_{z}\right)}^{2}}{2\sigma_{z}^{2}}\right)dxdzds \end{array}$$

and

(82) $$\begin{array}{cc} \; & \int_{\tau }^{t}\int_{M}\left(x^{2}\circ {\tilde{S}}^{s}\right)\Omega^{f_{0}}f_{0}\left(\Gamma \right)d\Gamma ds=\frac{1}{2\pi \sigma_{x}\sigma_{z}}\int_{\tau }^{t}\int_{M}[A\left(\tau \right)x-\frac{B\left(\tau \right)K_{2}}{2\sqrt{\gamma }}-\frac{B(\tau )z}{\sqrt{\gamma }}\\ \; & \;-\frac{\left(t-\tau \right)}{2\sqrt{\gamma }}\left(B\left(\tau \right)K_{1}+A\left(\tau \right)K_{2}\right))\cos\left(\sqrt{\gamma }s\right)+(B\left(\tau \right)x+\frac{A\left(\tau \right)K_{2}}{2\sqrt{\gamma }}-\frac{A(\tau )z}{\sqrt{\gamma }}\\ \; & \;+\frac{\left(t-\tau \right)}{2\sqrt{\gamma }}\left(A\left(\tau \right)K_{1}-B\left(\tau \right)K_{2}\right))\sin\left(\sqrt{\gamma }s\right){]}^{2}\left(\frac{\left(x-\mu_{x}\right)}{\sigma_{x}^{2}}z-\frac{\left(z-\mu_{z}\right)}{\sigma_{z}^{2}}\gamma x\right)\\ \; & \;\times \exp\left(-\frac{{\left(x-\mu_{x}\right)}^{2}}{2\sigma_{x}^{2}}-\frac{{\left(z-\mu_{z}\right)}^{2}}{2\sigma_{z}^{2}}\right)dxdzds\\ \; & =\frac{1}{2\pi \sigma_{x}\sigma_{z}}\int_{\tau }^{t}\int_{M}[\left(A\left(\tau \right)\cos\left(\sqrt{\gamma }s\right)+B\left(\tau \right)\sin\left(\sqrt{\gamma }s\right)\right)x\\ \; & \;-\left(A\left(\tau \right)\sin\left(\sqrt{\gamma }s\right)+B\left(\tau \right)\cos\left(\sqrt{\gamma }s\right)\right)\frac{z}{\sqrt{\gamma }}){]}^{2}\left(\frac{\left(x-\mu_{x}\right)}{\sigma_{x}^{2}}z-\frac{\left(z-\mu_{z}\right)}{\sigma_{z}^{2}}\gamma x\right)\\ \; & \;\times \exp\left(-\frac{{\left(x-\mu_{x}\right)}^{2}}{2\sigma_{x}^{2}}-\frac{{\left(z-\mu_{z}\right)}^{2}}{2\sigma_{z}^{2}}\right)dxdzds, \end{array}$$

while

(83) $$\begin{array}{cc} \; & \int_{\tau }^{t}\int_{\tilde{M}}\left(x^{2}\circ {\tilde{S}}^{s}\right)\left(A(\tau )\phi +B(\tau )\theta \right)\frac{\left(z-\mu_{z}\right)}{\sigma_{z}^{2}}{\tilde{f}}_{0}\left(\tilde{\Gamma }\right)d\tilde{\Gamma }ds\\ \; & =\frac{1}{2\pi \rho }\int_{M_{\theta \phi }}\left(A(\tau )\phi +B(\tau )\theta \right)d\theta d\phi \int_{\tau }^{t}\frac{1}{2\pi \sigma_{x}\sigma_{z}}\int_{M}\frac{\left(z-\mu_{z}\right)}{\sigma_{z}^{2}}[A\left(\tau \right)x-\frac{B\left(\tau \right)K_{2}}{2\sqrt{\gamma }}\\ \; & \;-\frac{B(\tau )z}{\sqrt{\gamma }}-\frac{\left(t-\tau \right)}{2\sqrt{\gamma }}\left(B\left(\tau \right)K_{1}+A\left(\tau \right)K_{2}\right))\cos\left(\sqrt{\gamma }s\right)+(B\left(\tau \right)x+\frac{A\left(\tau \right)K_{2}}{2\sqrt{\gamma }}-\frac{A(\tau )z}{\sqrt{\gamma }}\\ \; & \;+\frac{\left(t-\tau \right)}{2\sqrt{\gamma }}\left(A\left(\tau \right)K_{1}-B\left(\tau \right)K_{2}\right))\sin\left(\sqrt{\gamma }s\right){]}^{2}\exp\left(-\frac{{\left(x-\mu_{x}\right)}^{2}}{2\sigma_{x}^{2}}-\frac{{\left(z-\mu_{z}\right)}^{2}}{2\sigma_{z}^{2}}\right)dxdzds\\ \; & =\frac{1}{{\left(2\pi \right)}^{2}\rho \sigma_{x}\sigma_{z}}\int_{M_{\theta \phi }}\left(A(\tau )\phi +B(\tau )\theta \right)d\theta d\phi \int_{\tau }^{t}\int_{M}\frac{\left(z-\mu_{z}\right)}{\sigma_{z}^{2}}[\left(A\left(\tau \right)\cos\left(\sqrt{\gamma }s\right)+B\left(\tau \right)\sin\left(\sqrt{\gamma }s\right)\right)x\\ \; & \;-\left(A\left(\tau \right)\sin\left(\sqrt{\gamma }s\right)+B\left(\tau \right)\cos\left(\sqrt{\gamma }s\right)\right)\frac{z}{\sqrt{\gamma }}){]}^{2}\exp\left(-\frac{{\left(x-\mu_{x}\right)}^{2}}{2\sigma_{x}^{2}}-\frac{{\left(z-\mu_{z}\right)}^{2}}{2\sigma_{z}^{2}}\right)dxdzds. \end{array}$$

Concerning O=z instead, recalling that $E_{f_{0}}\left[z\right]=\mu_{z}$, one has

(84) $$\begin{array}{cc} \; & \int_{0}^{\tau }\int_{M}\left(z\circ S^{s}\right)\Omega^{f_{0}}f_{0}\left(\Gamma \right)d\Gamma ds=\frac{1}{2\pi \sigma_{x}\sigma_{z}}\int_{0}^{\tau }\int_{M}\left(-\sqrt{\gamma }x\sin\left(\sqrt{\gamma }s\right)\right)\left(\frac{\left(x-\mu_{x}\right)}{\sigma_{x}^{2}}z-\frac{\left(z-\mu_{z}\right)}{\sigma_{z}^{2}}\gamma x\right)\\ \; & \;\times \exp\left(-\frac{{\left(x-\mu_{x}\right)}^{2}}{2\sigma_{x}^{2}}-\frac{{\left(z-\mu_{z}\right)}^{2}}{2\sigma_{z}^{2}}\right)dxdzds=\mu_{z}\left(\cos\left(\sqrt{\gamma }t\right)-\cos\left(\sqrt{\gamma }\tau \right)\right) \end{array}$$

and

(85) $$\begin{array}{cc} \; & \int_{\tau }^{t}\int_{M}\left(z\circ {\tilde{S}}^{s}\right)\Omega^{f_{0}}f_{0}\left(\Gamma \right)d\Gamma ds=\frac{1}{2\pi \sigma_{x}\sigma_{z}}\int_{\tau }^{t}\int_{M}[(A\left(\tau \right)z_{\tau }-\frac{B\left(\tau \right)K_{1}}{2\sqrt{\gamma }}+B\left(\tau \right)x_{\tau }\sqrt{\gamma }\\ \; & \;+\frac{\left(s-\tau \right)}{2}\left(A\left(\tau \right)K_{1}-B\left(\tau \right)K_{2}\right))\cos\left(\sqrt{\gamma }s\right)+(B\left(\tau \right)z_{\tau }+\frac{A\left(\tau \right)K_{1}}{2\sqrt{\gamma }}-A\left(\tau \right)x_{\tau }\sqrt{\gamma }\\ \; & \;+\frac{\left(s-\tau \right)}{2}\left(A\left(\tau \right)K_{2}+B\left(\tau \right)K_{1}\right))\sin\left(\sqrt{\gamma }s\right)]\left(\frac{\left(x-\mu_{x}\right)}{\sigma_{x}^{2}}z-\frac{\left(z-\mu_{z}\right)}{\sigma_{z}^{2}}\gamma x\right)\\ \; & \;\times \exp\left(-\frac{{\left(x-\mu_{x}\right)}^{2}}{2\sigma_{x}^{2}}-\frac{{\left(z-\mu_{z}\right)}^{2}}{2\sigma_{z}^{2}}\right)dxdzds\\ \; & =\frac{1}{2\pi \sigma_{x}\sigma_{z}}\int_{\tau }^{t}\int_{M}[\left(A\left(\tau \right)\cos\left(\sqrt{\gamma }s\right)+B\left(\tau \right)\sin\left(\sqrt{\gamma }s\right)\right)z_{\tau }+(B\left(\tau \right)\sqrt{\gamma }\cos\left(\sqrt{\gamma }s\right)\\ \; & \;-A\left(\tau \right)\sqrt{\gamma }\sin\left(\sqrt{\gamma }s\right))]\left(\frac{\left(x-\mu_{x}\right)}{\sigma_{x}^{2}}z-\frac{\left(z-\mu_{z}\right)}{\sigma_{z}^{2}}\gamma x\right)\exp\left(-\frac{{\left(x-\mu_{x}\right)}^{2}}{2\sigma_{x}^{2}}-\frac{{\left(z-\mu_{z}\right)}^{2}}{2\sigma_{z}^{2}}\right)dxdzds, \end{array}$$

while

(86) $$\begin{array}{cc} \; & \int_{\tau }^{t}\int_{\tilde{M}}\left(z\circ {\tilde{S}}^{s}\right)\left(A(\tau )\phi +B(\tau )\theta \right)\frac{\left(z-\mu_{z}\right)}{\sigma_{z}^{2}}{\tilde{f}}_{0}\left(\tilde{\Gamma }\right)d\tilde{\Gamma }ds\\ \; & =\frac{1}{2\pi \rho }\int_{M_{\theta \phi }}\left(A(\tau )\phi +B(\tau )\theta \right)d\theta d\phi \int_{\tau }^{t}\frac{1}{2\pi \sigma_{x}\sigma_{z}}\int_{M}\frac{\left(z-\mu_{z}\right)}{\sigma_{z}^{2}}[(A\left(\tau \right)z_{\tau }-\frac{B\left(\tau \right)K_{1}}{2\sqrt{\gamma }}+B\left(\tau \right)x_{\tau }\sqrt{\gamma }\\ \; & \;+\frac{\left(t-\tau \right)}{2}\left(A\left(\tau \right)K_{1}-B\left(\tau \right)K_{2}\right))\cos\left(\sqrt{\gamma }t\right)+(B\left(\tau \right)z_{\tau }+\frac{A\left(\tau \right)K_{1}}{2\sqrt{\gamma }}-A\left(\tau \right)x_{\tau }\sqrt{\gamma }\\ \; & \;+\frac{\left(t-\tau \right)}{2}\left(A\left(\tau \right)K_{2}+B\left(\tau \right)K_{1}\right))\sin\left(\sqrt{\gamma }s\right)]\exp\left(-\frac{{\left(x-\mu_{x}\right)}^{2}}{2\sigma_{x}^{2}}-\frac{{\left(z-\mu_{z}\right)}^{2}}{2\sigma_{z}^{2}}\right)dxdzds\\ \; & =\frac{1}{{\left(2\pi \right)}^{2}\rho \sigma_{x}\sigma_{z}}\int_{M_{\theta \phi }}\left(A(\tau )\phi +B(\tau )\theta \right)d\theta d\phi \int_{\tau }^{t}\int_{M}\frac{\left(z-\mu_{z}\right)}{\sigma_{z}^{2}}[\left(A\left(\tau \right)\cos\left(\sqrt{\gamma }s\right)+B\left(\tau \right)\sin\left(\sqrt{\gamma }s\right)\right)z_{\tau }\\ \; & \;+(B\left(\tau \right)\sqrt{\gamma }\cos\left(\sqrt{\gamma }s\right)-A\left(\tau \right)\sqrt{\gamma }\sin\left(\sqrt{\gamma }s\right))]\exp\left(-\frac{{\left(x-\mu_{x}\right)}^{2}}{2\sigma_{x}^{2}}-\frac{{\left(z-\mu_{z}\right)}^{2}}{2\sigma_{z}^{2}}\right)dxdzds. \end{array}$$

To better understand the time evolution of such observables, we choose to consider a wider time interval compared to the one introduced at the beginning of the section. For the sake of this representation, we considered the case where system (62) is defined for t≤10τ, meaning that the piecewise memory kernel has been picked as γ(t−s)=γ for t∈(τ,10τ] and 0 otherwise. Initial conditions at t=τ have been set in order to guarantee continuity and differentiability of the trajectory coming from the interval t∈(0,τ], meaning that $x_{\tau }=x_{0}\cos\left(\sqrt{\gamma }\tau \right)$ and $z_{\tau }=-\sqrt{\gamma }x_{0}\sin\left(\sqrt{\gamma }\tau \right)$.

We remark that the method we showed for a two-step kernel can be iterated for a bigger number of steps thus approximating more complex functional forms. This approach suggests an algorithmic scheme suitable for treating the problems numerically, whose point of view is often necessary for a wide variety of phenomena [39,40].

## 4. Comparison Between Linear Response and Exact Response

Let us now show how the exact response method performs against a linear response: for the sake of simplicity, we consider the memory kernel for the two steps and we restrict to the case where $\mu_{v}=0$, $\mu_{p}=0$, $\sigma_{v}^{2}=1/\beta \gamma$ and $\sigma_{p}^{2}=1/\beta$. Let us consider a perturbation of a simple Hamiltonian, whose variables of position and momentum are denoted by (v, p):

(87) $$\begin{array}{cc} H(v,p,t) & =\frac{1}{2}p^{2}+\gamma \frac{1}{2}v^{2}-w\left(t\right)v\\ \; & =H_{0}\left(v,p\right)+H_{p}\left(v,p,t\right), \end{array}$$

where $H_{0}\left(v,p\right):=\frac{1}{2}p^{2}+\gamma \frac{1}{2}v^{2}$ is the unperturbed Hamiltonian describing the unperturbed dynamics of the harmonic oscillator of (61) and $H_{p}\left(v,p,t\right):=-w\left(t\right)v=-v\left(A\left(\tau \right)\cos\left(\sqrt{\gamma }t\right)+B\left(\tau \right)\sin\left(\sqrt{\gamma }t\right)\right)$ corresponds to the perturbation that starts acting on the system at time t=τ. Within this formalism, (v, p) represent the coordinates in the symplectic space in which *H* is defined and are physically equivalent to (x, z) supposing the “mass” is set to 1.

Under these assumptions, the response function (8) is a two-time equilibrium correlation function, in which only the unperturbed dynamics is used,

(88) $$R\left(t\right)=-\beta E_{f_{0}}\left[\left(O\circ S_{0}^{s}\right)\dot{\pi }\left(\Gamma \right)\right]$$

and since this is for a Hamiltonian dynamics $J(\Gamma )=-\frac{d\pi (\Gamma )}{dt}$, the linear response Equation (7) takes the following form:

(89) $$\;E_{f_{t}}^{\left(lin\right)}\left[O\right]=E_{f_{0}}\left[O\right]+\beta \int_{0}^{t}w\left(s\right)E_{f_{0}}\left[\left(O\circ S_{0}^{s}\right)\cdot \dot{\pi }\right]ds,$$

in which $\beta =\frac{1}{k_{B}T}$ and $f_{0}\left(\Gamma \right)=e^{-\beta H_{0}\left(\Gamma \right)}/Z$ is the canonical equilibrium distribution.

The unperturbed flow $S_{0}^{s}$ considers the dynamics before the perturbation acts on the system at time τ and therefore follows from the equations of motion of $H_{0}$ for the variables (v, p).

We now focus on comparing the linear response with the exact response for three observables, *v*, *p* and $v^{2}$.

The linear response for O=v reads as

(90) $$E_{f_{t}}^{\left(lin\right)}\left[v\right]=E_{f_{0}}\left[v\right]+\beta \int_{0}^{t}w\left(s\right)E_{f_{0}}\left[\left(v\circ S_{0}^{s}\right)\cdot \dot{\pi }\right]ds.$$

Direct calculations leads to

(91) $$E_{f_{t}}^{\left(lin\right)}\left[v\right]=0.$$

**Figure 1:**
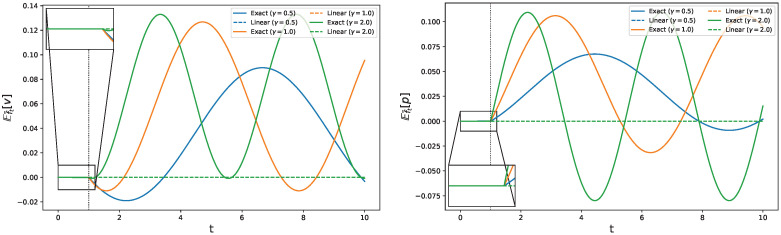
The left (right) panel shows the comparison in the evolution of the response of observable O=x, indicated as *v* in the linear picture (O=z, replaced by *p* in the linear picture), between exact (continuous) and linear (dotted) frameworks, for different values of the forcing frequency: γ=0.5 (blue), γ=1.0 (orange) and γ=2.0 (green). For t∈[0,τ), the dynamics are linear, and the linear and exact response coincide, as evidenced in the magnification boxes. At t=τ, the kernel γ changes discontinuously, which amounts to a strong nonlinearity. The linear response does not capture this effect, the exact one does: the response result is continuous, with a discontinuity in its time derivative.

The evolution of *v* according to the exact dynamics is compared graphically with the evolution to the linear one and showed in the left panel of Figure 1.

O=p instead has a linear response that evolves according to

(92) $$E_{f_{t}}^{\left(lin\right)}\left[p\right]=E_{f_{0}}\left[p\right]+\beta \int_{0}^{t}w\left(s\right)E_{f_{0}}\left[\left(p\circ S_{0}^{s}\right)\cdot \dot{\pi }\right]ds,$$

which is equal to

(93) $$E_{f_{t}}^{\left(lin\right)}\left[p\right]=0$$

The same comparison for the *p* variable is represented in the right panel of Figure 1.

Finally, regarding the observable $O=v^{2}$, direct calculation leads to a vanishing response, i.e.,

(94) $$E_{f_{t}}^{\left(lin\right)}\left[v^{2}\right]=E_{f_{0}}\left[v^{2}\right]=\frac{1}{\beta \gamma }.$$

The linear response theory leads to a time-constant term, which is a strong approximation of the actual dynamics, as proven in the previous section.

For a valid comparison between the linear and exact responses, computed for velocity, acceleration and squared velocity, we set the parameters of the equilibrium distribution $f_{0}$, involved in the exact response calculation, to match the ones of the canonical distribution involved in the linear approach. The following pictures, comparing the evolution of the response of different observables, refer then to $\mu_{x}=\mu_{z}=0$, $\sigma_{x}^{2}=1/\beta \gamma$ and $\sigma_{z}^{2}=1/\beta$ with β=1000 in order to ensure that the variance $\sigma_{z}$ is small enough to approximate a Dirac delta centered on $z_{0}=0$.

While the linear formalism (dotted lines) predicts a null response for variables *v* and *p*, Figure 1 shows how, within the exact formalism (continuous lines), both observables present oscillations whose amplitude and frequency depend on the specific choice of γ. Moreover, it is interesting to notice how the oscillation amplitudes increase consistently with increasing values of γ, despite the non-linear dependence on the forcing frequency. Indeed, such a parameter is present not only as the frequency of the forcing, but also alongside $x_{0}$ as a multiplying factor in the dynamics (68), influencing both the frequency and amplitude of oscillations at the same time. The accordance between the linear and exact regimes is perfectly captured for 0<t<τ=1, where the system is described by the unperturbed dynamics (61). Since the time-dependent forcing w(t) is delayed by a factor τ, differences between the two responses emerge from t≥τ when a strong nonlinearity (a discontinuity in the kernel γ) is switched on. Indeed, the linear form of the observables, the piecewise constant form of γ, and the symmetry of the initial distribution prevent the linear response from realizing that the dynamics changes at t=τ.

**Figure 2:**
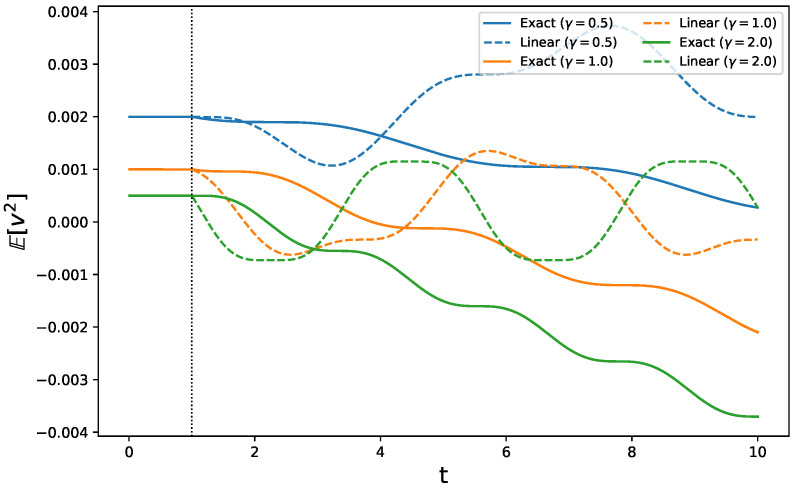
Comparison of the predicted response for observable $O=v^{2}$ between the exact response (continuous line) and the linear regime (dotted line) for different values of the forcing frequency: γ=0.5 (blue), γ=1.0 (orange) and γ=2.0 (green). For t∈[0,τ), the linear and exact response coincide, as expected. For t>τ, the equivalence of the two responses is lost.

Concerning variable $x^{2}$ ($v^{2}$ in the linear framework), displayed in Figure 2, it is interesting to notice how, within the linear regime, such a response exhibits oscillatory patterns that differ from the value of γ. The exact response shows instead an overall decreasing behavior with oscillations of bigger amplitudes the greater the value of γ. In this case, the observable is sufficiently sensitive to the perturbation, and even its linear response evolves in time for t>τ. It differs, however, from the exact response.

Finally, we note that we have treated a case in which the initial value of *z* equals zero, according to Equation (23). This can be easily generalized by adding the constant $z_{0}$ to the right-hand side of that equation. The response can then be computed for an initial distribution of $z_{0}$ values. If this distribution has a mean of 0, the present case can be recovered in the limit in which its variance shrinks to 0.

## 5. Conclusions

In this work we showed how the applicability of the exact response theory developed in the field of molecular dynamics [7] extends to delay equations. The approach followed the ideas first developed by [17,18], for time-dependent and stochastic dynamics, based on auxiliary variables that eliminate the explicit time dependence of perturbations, without affecting the phase-space variation rate.

We focused on simple examples to illustrate how this can be done. One needs the delay equation to be cast in a set of ODEs, whose solution must be computed as normal. Once this solution is available, the machinery of the response theory allows to effectively compute the statistics of all observables of interest, including the evolution of their uncertainty. As a first example we have an exponentially decaying memory kernel, which is common, for example, for viscoelasticity [38]. This model is equivalent to autonomous dynamics, with viscosity or friction, and can thus be tackled directly with the exact response theory. Naturally, this is not always the case. Subsequently, we focused on a more general case, a second-order Erlang-type kernel that exhibits non monotonic memory behavior, typically employed in many problems in the context of anomalous transport.

As a third example, we thus focused on kernels that are piecewise constant, i.e., belong to the class of simple functions, dense e.g., in $L^{1}$, and hence capable of approximating to any desired accuracy a very wide class of kernels. An interesting feature of these kernels is resonant forcing, that acts on a harmonic oscillator. The oscillatory behavior of the forcing allows a convenient parameterization of the dynamics in the augmented phase space. The resulting dynamics is physically equivalent to the original one, but autonomous, making the exact response theory applicable.

It is important to note that the auxiliary dynamics in $M_{\theta \phi }$ does not alter the dynamics. However, it comes with the benefit of one further degree of randomness, or of uncertainty, that can be profitably quantified and used in practical applications. Indeed, not only the initial conditions of the system of interest are usually only partially known, but also the actions of the perturbing agents. The choice of a δ-distributed initial condition for the augmented phase-space variables $\left(\theta_{0},\phi_{0}\right)$ therefore not only does not satisfy the ergodic consistency but would also not be achievable as fixing specific initial conditions $\theta_{0}$, $\phi_{0}$ would not be desirable, being that the initial perturbation, like the initial conditions in general, cannot be known with infinite accuracy. The comparison with the linear response framework emphasizes the generality of the exact response machinery in capturing more complex behaviors beyond the linear regime. Our work in this respect is introductory, but clarifies a method for computing the response of observables of systems characterized by time-delay satisfying integro-differential equations of the kind (23). These constitute a vast and important branch of current research that is impossible to summarize here, see Refs. [41,42].

Further considerations must be performed: the applicability of this method strongly relies on the existence and uniqueness of the solutions of the dynamical system in the restricted phase space M, which is not always guaranteed. In addition, the construction of the piecewise kernel model is complex and computationally demanding.

Possible directions to be pursued in future research include exploring the applicability of the exact response theory to other kinds of time-delay equations, not necessarily in the form of integro-differential equations. 

## Data Availability

The original contributions presented in this study are included in the article. Further inquiries can be directed to the corresponding author.

## Appendix A. Alternative Proof for the Piecewise Kernel

An alternative derivation of the dynamics governing the system with a stepwise continuous memory kernel (58) employs careful integration steps depending on the adopted discretization. Starting from t∈(0,τ], we have

(A1) $$\dot{v}\left(t\right)=-\gamma_{1}\int_{0}^{t}v\left(s\right)ds$$

and differentiating we obtain

(A2) $$\ddot{v}\left(t\right)+\gamma_{1}v\left(t\right)=0,$$

that represents a harmonic oscillator with frequency $\omega =\sqrt{\gamma_{1}}$. Moving to t∈(τ,2τ], a second memory step $\gamma_{2}$ joins the dynamics, with $0<\gamma_{2}<\gamma_{1}$. Integrating, one obtains

(A3) $$\dot{v}\left(t\right)=-\gamma_{2}\int_{0}^{t-\tau }v\left(s\right)ds-\gamma_{1}\int_{t-\tau }^{t}v\left(s\right)ds$$

and differentiating again leads to

(A4) $$\ddot{v}\left(t\right)+\gamma_{1}v\left(t\right)=-\left(\gamma_{2}-\gamma_{1}\right)v\left(t-\tau \right),$$

with $v\left(t-\tau \right)=v_{0}\cos\left(\sqrt{\gamma_{1}}\left(t-\tau \right)\right)$, representing a forced harmonic oscillator in resonance. One can now continue, successively joining and keeping continuity and differentiability, the solutions of equations of this form.

## Appendix B. Alternative Choices for *g*_0_

The choice of $g_{0}$ is arbitrary and free, since the dynamics must not be dependent on the fictitious dynamics. Let us take as a matter of example the sine variant of the bivariate von Mises distribution [43]:

(A5) $$g_{0}\left(\theta ,\phi \right)=Z_{s}\left(k_{1},k_{2},k_{3}\right)\exp\left(k_{1}\cos\left(\theta -\mu \right)+k_{2}\cos\left(\phi -\nu \right)+k_{3}\sin\left(\theta -\mu \right)\sin\left(\phi -\nu \right)\right),$$

where μ, ν are the means for θ, ϕ, respectively, and $k_{1},\;k_{2}$ are their concentrations and $k_{3}$ is related to their correlation.

Applying this distribution to the stepwise constant function, the third term for ${\bar{\Omega }}^{{\tilde{f}}_{0}}$ is no longer 0 but is equals to

(A6) $$\begin{array}{cc} \; & -\frac{1}{g_{0}\left(\theta ,\phi \right)}\left(\phi \frac{\partial g_{0}(\theta ,\phi )}{\partial \theta }-\theta \frac{\partial g_{0}(\theta ,\phi )}{\partial \phi }\right)\\ = & -k_{1}\phi \sin\left(\theta -\mu \right)-k_{2}\theta \sin\left(\phi -\nu \right)+k_{3}\left[\theta \sin\left(\theta -\mu \right)\cos\left(\phi -\nu \right)-\phi \sin\left(\phi -\nu \right)\cos\left(\theta -\mu \right)\right]. \end{array}$$

When the term (A6) is different from 0, then (22) takes an extra term of the form

(A7) $$-E_{f_{0}}\left[O\circ S^{s}\Gamma \right]\int_{M_{\theta \phi }}d\theta d\phi \left(\phi \frac{\partial g_{0}}{\partial \theta }-\theta \frac{\partial g_{0}}{\partial \phi }\right).$$

As precisely explained in [18], this term is equal to 0 due to the interchangeable role of the auxiliary variables.

This argument, which here has been carried out using a common known distribution, holds for any choice of differentiable $g_{0}$. For simplicity, it is often sufficient to use the uniform distribution, which simplifies calculations.
